# RNA Modifications in Gastrointestinal Cancer: Current Status and Future Perspectives

**DOI:** 10.3390/biomedicines10081918

**Published:** 2022-08-08

**Authors:** Xiaoting Zhang, Hao Su, Hongyan Chen, Qing Li, Xiaodong Liu, Lin Zhang, William Ka Kei Wu, Matthew Tak Vai Chan, Huarong Chen

**Affiliations:** 1 Department of Anaesthesia and Intensive Care and Peter Hung Pain Research Institute, The Chinese University of Hong Kong, Hong Kong 999077, China; 2 CUHK Shenzhen Research Institute, Shenzhen 518000, China; 3 Li Ka Shing Institute of Health Sciences, The Chinese University of Hong Kong, Hong Kong 999077, China; 4 State Key Laboratory of Digestive Diseases, The Chinese University of Hong Kong, Hong Kong 999077, China

**Keywords:** gastrointestinal cancer, RNA modification, therapeutic target

## Abstract

Gastrointestinal (GI) cancer, referring to cancers of the digestive system such as colorectal cancer (CRC), gastric cancer (GC), and liver cancer, is a major cause of cancer-related deaths in the world. A series of genetic, epigenetic, and epitranscriptomic changes occur during the development of GI cancer. The identification of these molecular events provides potential diagnostic, prognostic, and therapeutic targets for cancer patients. RNA modification is required in the posttranscriptional regulation of RNA metabolism, including splicing, intracellular transport, degradation, and translation. RNA modifications such as N6-methyladenosine (m6A) and N1-methyladenosine (m1A) are dynamically regulated by three different types of regulators named methyltransferases (writers), RNA binding proteins (readers), and demethylases (erasers). Recent studies have pointed out that abnormal RNA modification contributes to GI tumorigenesis and progression. In this review, we summarize the latest findings on the functional significance of RNA modification in GI cancer and discuss the therapeutic potential of epitranscriptomic inhibitors for cancer treatment.

## 1. Introduction

Gastrointestinal (GI) cancers refer to malignant conditions of the GI tract and accessory organs of digestion. Globally, GI cancers account for roughly half of all cancer-related deaths [1,2]. Although conventional therapy has been successful to some extent, drug resistance and cancer recurrence are still major obstacles during cancer treatment. Emerging evidence has suggested that abnormal RNA modifications play a pivotal role during the development of GI cancers and may serve as potential therapeutic targets for cancer patients.

**Figure 1:**
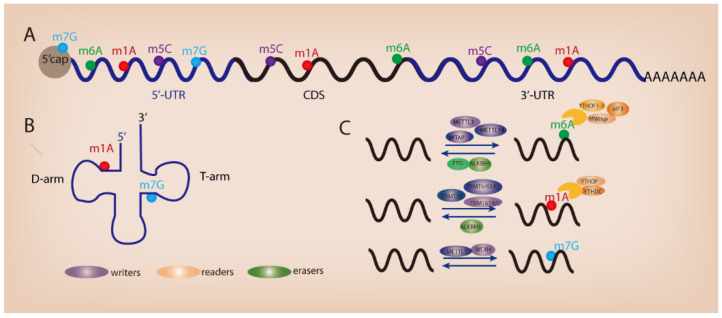
**Different types of RNA modifications and their distribution.** (**A**) RNA modifications within mRNA. (**B**) RNA modifications in tRNA. (**C**) Regulators of RNA modifications.

The advent of high-throughput sequencing technology allows the high-precision mapping of different types of RNA modifications. Since the discovery of the first RNA nucleoside modification 60 years ago [3], over 170 different types of post-transcriptional modifications have been identified in primary RNA transcripts such as messenger RNAs (mRNAs), ribosomal RNAs (rRNAs), and transfer RNAs (tRNAs) [4]. RNA modifications have been recognized as crucial regulators of gene expression across time and space in eukaryotic cells. The presence of RNA modifications serves as a key switch on RNA metabolism and remarkably influences the process of RNA splicing, trafficking, stability, and translation efficiency [5,6]. To date, several widespread RNA modifications have been revealed in mammalian RNAs such as N6-methyladenosine (m6A) [7,8], N1-methyladenosine (m1A) [9,10], 5-methylcytosine (m5C) [11,12], and N7-methylguanosine (m7G) (Figure 1A,B). Similar to DNA epigenetics and histone modifications, RNA modifications can be installed, removed, and decoded by specific proteins (known as “writers”, “erasers”, and “readers”) (Figure 1C). RNA modifications play an important role in various biological processes. Dysregulated RNA modifications contribute to tumorigenesis and tumor progression through modulating cell differentiation, growth, survival, migration, and chemo-drug resistance [13]. In this review, we describe the functional importance of RNA m6A, m1A, and m7G modifications in GI cancer and discuss their potential role as therapeutic targets for cancer patients.

## 2. Common Types of Modifications in RNA

### 2.1. N6-Methyladenosine

N6-methyladenosine (m6A), referring to the methylation of the adenosine base at the nitrogen-6 position, is the most prevalent internal mRNA modification [14]. m6A modification is dynamic and reversible and is involved in almost all processes of mRNA metabolism, including RNA transcription, translation, and degradation [15,16,17]. The enzymes catalyzing the formation of m6A are named as m6A writers, including METTL3, METTL14, and WTAP [18,19]. On the other hand, m6A markers can be removed by m6A erasers such as FTO [20,21,22] and ALKBH5 [23,24]. In addition, m6A can be recognized by a set of RNA-binding proteins called m6A readers that can specifically recognize and bind to m6A-modified transcripts, e.g., YTHDF1/2/3 and YTHDC1/2 (the YTH domain family proteins) [25,26,27,28,29], IGF2BP1/2/3 (the insulin-like growth factor 2 mRNA-binding proteins) [30], and hnRNPC/hnRNPG (the heterogeneous nuclear ribonucleoproteins), leading to the change of RNA metabolism [31,32].

### 2.2. N1-Methyladenosine

N1-methyladenosine (m1A) refers to the methylation of adenosine at position 1. In human tRNAs, m1A modification is commonly observed at the conserved nucleotide 58 in the TΨC loop, which is critical for tRNA stability [33]. Moreover, a recent study revealed hundreds of m1A sites in human mRNA and long noncoding RNAs (lncRNAs) by mapping m1A at single-nucleotide resolution, although present with low stoichiometry [34]. The occurrence of m1A sites in the 5′ untranslated region (5′ UTR) or coding sequence (CDS), but not 3′UTR, of mRNA, was found to be associated with translational repression [34], implying that m1A enrichment in mRNA may affect the process of ribosomal scanning. Consistent data were obtained by another research team showing that a large number of m1A modification sites appear in human mRNA [35]. The m1A writers (TRMT10C, Trmt61B, and TRMT6/61A), readers (YTHDF1, YTHDF2, YTHDF3, and YTHDC1), and erasers (ALKBH1, ALKBH3) regulate the process of m1A modifications and have a robust role in posttranscriptional regulation of m1A-modified mRNAs and ncRNAs [34,35,36,37]. Trmt61B and TRMT6/61A catalyze m1A at position 58 of mt and cyt tRNA in humans; on the other hand, TRMT10C catalyzes m1A at position 9 [34,37,38]. In addition, ALKBH1 and ALKBH3 demethylate m1A marks in single-stranded (ss) DNA and RNA [39,40]. Moreover, YTH domain-containing proteins, such as YTHDF1, YTHDF2, YTHDF3, and YTHDC1, directly bind to m1A-bearing RNA to trigger the subsequent RNA metabolism process [41].

### 2.3. N7-Methylguanosine

N7-methylguanosine (m7G) refers to the methylation of guanosine on position N7 of RNA. It is commonly observed in the tRNA variable loop and mRNA 5′ Cap region [42,43]. Extensive m7G tRNA or mRNA methylomes have been identified in mammals [43,44,45,46]. METTL1-WDR4 is the methyltransferase complex that induces m7G modification, leading to the change in RNA metabolism such as mRNA translation [42,43]. Accumulating evidence has pointed out the occurrence of aberrant m7G modification during human disease development, especially in cancer. Dysregulated m7G modification could contribute to tumor formation and progression through regulating the expression of downstream oncogenes or tumor suppressor genes.

### 2.4. RNA Modification and RNA Metabolism

RNA modification plays an important role in post-transcriptional regulation. It could alter RNA splicing, regulate RNA export, and tune RNA stability and translation via the change of RNA secondary structures, RNA–RNA, and RNA–protein interactions [47,48]. Accumulating evidence suggests that m6A modifications control pre-mRNA processing. The co-localization of m6A regulators such as the m6A writers, METTL3, METTL14, and WTAP, as well as m6A erasers, ALKBH5 and FTO, with splicing factors, has been identified in the nuclear speckles. The occurrence of m6A marks could impact the binding capacity of splicing factors, thereby influencing the alternative splicing of targeted genes [18,49,50]. Conversely, the removal of m6A by S-adenosylmethionine (SAM) synthesis inhibitors, neplanocin A or cycloleucine, led to the nuclear accumulation of unspliced transcripts [51,52]. Recent work has also pointed out that m6A modifications regulate RNA export. The m6A methyltransferases (METTL3, METTL14, WTAP, and KIAA1429) could bind to the TRanscription-Export (TREX) complex which plays a major role in RNA nuclear export [53].

M6A modifications also control RNA stability and translation. YTHDF2 is recognized as a major decay-inducing m6A reader protein through binding to m6A-modified mRNAs and recruiting RNA-degrading enzymes [54,55]. To date, there are two distinct mechanisms of YTHDF2-induced mRNA degradation being reported: the RNase P/MRP-mediated endoribonucleolytic-cleavage pathway and the carbon catabolite repression 4 (CCR4)-negative on TATA-less (NOT)-mediated deadenylation pathway [56]. As to m6A-mediated mRNA translation, different mechanisms have been revealed. The m6A reader YTHDF1 is known to bind to m6A-modified mRNA and recruit the initiation factor eukaryotic initiation factor 3 (eIF3), resulting in increased ribosome occupancy at the mRNA that facilitates translation [27]. It is worth noting that METTL3 could promote mRNA translation in both m6A-dependent and -independent manner [57,58,59,60]. In addition to its role as an m6A writer, METTL3 at 3′UTR near the stop codon could directly interact with eIF3h at the 5′ UTR of the mRNA to form a loop that drives ribosome recycling [60].

RNA m6A modifications are required for DNA damage repair. In response to ultraviolet irradiation, m6A modification was rapidly induced in RNA at DNA damage sites, which was regulated by METTL3 and FTO [61]. Subsequently, m6A RNA recruited Pol κ to DNA damage sites to facilitate repair and cell survival [61]. In agreement, METTL3 and YTHDC1 are reported to regulate the homologous recombination (HR)-mediated repair of DNA double-strand breaks (DSBs) [62]. Upon DSBs, phosphorylated METTL3 induced by ATM (ATM Serine/Threonine Kinase) translocated to DNA damage sites to catalyze m6A modification in RNAs which then recruit YTHDC1 and modulate DNA-RNA hybrids accumulation, resulting in recruitment of RAD51 and BRCA1 for HR-mediated repair [62].

M1A and m7G contain positive electrostatic charge under physiological conditions which could trigger electro-chemical interaction that is critical for tRNA structure and function [63]. M1A modification can disrupt the normal Watson–Crick base pairing of A: T or A: U to form Hoogsteen base pairs [9,10]. This would lead to the change of RNA structure, specific RNA–RNA or RNA–protein interactions, and eventually, RNA stability and mRNA translation [9,10]. Intriguingly, in response to multiple stimuli, m1A modifications display tissue specificity and cellular plasticity in mammals. M7G cap modification is evolutionarily conserved in eukaryotic mRNA. It is present during the early stages of RNA transcription induced by RNA polymerase II (Pol II). The m7G cap could be recognized by Cap Binding Complex (CBC), resulting in altered mRNA transcription, splicing, export, stability, and translation [45].

## 3. The Role of RNA Modification in GI Cancer

Recent studies have shown that RNA modification is closely associated with the development of gastrointestinal cancer, including liver cancer, colorectal cancer (CRC), and gastric cancer (GC). Herein, we summarize the recent findings of RNA modification in gastrointestinal cancer (Table 1, Table 2 and Table 3).

### 3.1. Liver Cancer

Liver cancer is the fifth most common cancer-related death. Epitranscriptomic alterations including RNA modification have been identified in liver cancer which plays an important role in liver cancer development [94,95].

METTL3 was reported to promote liver cancer progression; mechanistically, METTL3 increased SOCS2 mRNA m6A abundance, leading to SOCS2 mRNA degradation, which was mediated by YTHDF2 [64]. In hepatocellular carcinoma (HCC), METTL3 was SUMOylated by a small ubiquitin-like modifier SUMO1, which in turn facilitated the oncogenic function of METTL3 [65]. Notably, the SUMOylation of METTL3 could regulate mRNA homeostasis of Snail, a key transcription factor of EMT, in HCC [65]. In line with this study, abundant m6A modification was identified in Snail mRNA by m6A-sequencing [66]. Intriguingly, m6A enrichment in Snail CDS, but not 3′UTR, was associated with increased Snail mRNA translation, which was mediated by YTHDF1 in liver cancer [66]. The knockdown of METTL3 or YTHDF1 attenuated Snail expression and suppressed liver cancer migration and invasion [66]. Moreover, upregulated METTL3 and YTHDF1 expression was found in liver cancer that could serve as adverse prognosis factors for patients [66]. The m6A modifications are also enriched in Circular RNAs (circRNA). Circular cleavage and polyadenylation specific factor 6 (circCPSF6) is a newly identified m6A-modified circRNA. The depletion of ALKBH5 increased the m6A level of circCPSF6, leading to YTHDF2-mediated RNA destabilization [68]. Upregulated circCPSF6 expression in HCC promoted tumorigenicity and metastasis [68]. The m6A demethylase FTO expression was overexpressed in HCC, and the high expression of FTO was associated with a poor outcome in HCC patients [67]. Functionally, the depletion of FTO reduced HCC growth both in vitro and in vivo; mechanistically, FTO decreased the m6A abundance of PKM2 mRNA and promoted its expression in HCC [67].

The m6A modifications are also essential for liver cancer stemness. The knockdown of YTHDF2 could significantly inhibit the tumor-initiating ability of CD133+ liver cancer stem cells, while the overexpression of YTHDF2 exerted the opposite effect [69]. Mechanistically, YTHDF2 bound to m6A-modified OCT4 mRNA and increased its translation in liver cancer [69]. The ectopic expression of OCT4 was capable to restore the impaired stemness caused by YTHDF2 depletion, suggesting that YTHDF2 induced m6A-OCT4 to promote liver cancer stemness [69].

The role of m1A modification in liver cancers remains unclear. A recent study reported that RNA m1A level was induced in HCC tumors and liver cancer stem cells (CSCs) compared to adjacent normal tissues and that high m1A content predicted poor HCC patient survival [70]. In this study, the high expression of m1A methyltransferase complex TRMT6/TRMT61A in HCC could elevate the m1A methylation of tRNA, leading to increased PPARδ translation which in turn activated cholesterol synthesis and subsequent Hedgehog signaling, eventually driving liver tumorigenesis [70]. Therefore, TRMT6/TRMT61A-midiated m1A modification is essential for HCC development.

Accumulating evidence indicates an important role of m7G in various human disease development, especially cancer. A higher m7G tRNA modification level, as well as the upregulated expression of METTL1 and WDR4, two m7G methyltransferases, were observed in intrahepatic cholangiocarcinoma (ICC) compared to adjacent normal tissues [71]. Both in vitro and in vivo loss- and gain-of-function assays pointed out that METTL1/WDR4 promoted ICC cell survival and progression [71]. Mechanistically, METTL1/WDR4-induced m7G tRNA modification enhanced the mRNA transcription of oncogenes involved in cancer-related pathways such as the cell cycle and EGFR pathways [71]. Consistently, an elevated METTL1/WDR4 expression and an m7G tRNA modification level were identified in HCC compared to adjacent normal tissues, and METTL1/WDR4 exerted an oncogenic role in promoting HCC through m7G tRNA modification-dependent translation [72]. The oncogenic role of WDR4 in HCC was also reported by other research teams revealing that high WDR4 expression promoted HCC cell cycle progression, inhibited cell apoptosis, and increased HCC metastasis and sorafenib resistance [74]. Notably, METTL1 could modulate the immunosuppressive immune microenvironment in HCC. METTL1 expression was capable of inducing an accumulation of PMN-MDSCs which in turn inhibited CD8+ T cell infiltration, thereby facilitating HCC progression after insufficient radiofrequency ablation [73].

### 3.2. Colorectal Cancer

Colorectal cancer (CRC) is a leading cause of cancer morbidity and mortality around the world, and the development of CRC is regulated by genetics and epigenetic and epitranscriptomic mechanisms [96]. By integrative m6A sequencing, RNA sequencing, ribosome profiling, RNA immunoprecipitation sequencing, and proteomics, our team has recently identified a novel oncogenic epitranscriptome axis of METTL3-m6A-GLUT1-mTORC1 [75] and YTHDF1-m6A-ARHGEF2 [79] in promoting CRC tumorigenesis. The elevated expression of METTL3 in CRC could increase the m6A level of GLUT1 and promote its mRNA translation, leading to higher glucose uptake and lactate production which subsequently activated mTORC1 signaling [75]. On the other hand, YTHDF1 could directly bind to m6A-modified ARHGEF2 mRNA and enhance its translation, resulting in the subsequent activation of RhoA-signaling that enhanced CRC growth and metastasis [79]. Therefore, m6A modifications play a pivotal role in facilitating CRC tumorigenesis and progression. Intriguingly, Wnt-signaling in intestinal stem cells (ISCs) could induce YTHDF1 expression to promote the translation of TCF7L2/TCF4, inferring a positive feedback loop between YTHDF1-mediated m6A and Wnt signaling that promoted cancer stemness [97]. Consistently, increased YTHDF2 activity induced the Wnt/β-catenin pathway to promote CRC growth [80].

M6A modification is also critical for CRC metastasis. METTL3 can upregulate the m6A level of pri-miR-1246 to promote its maturation, thereby promoting CRC migration and invasion both in vitro and in vivo [76]. Moreover, YTHDC1 could bind to m6A-modified circNSUN2 to facilitate its export from the nucleus to the cytoplasm where cytoplasmic circNSUN2 formed an RNA-protein ternary complex with Insulin-Like Growth Factor 2 mRNA-Binding Protein 2 (IGF2BP2), high mobility group AT-hook 2 (HMGA2) mRNA and RNA-binding protein (RBP), resulting in the enhanced stability of HMGA2 which further promoted CRC liver metastasis [81].

m6A modification could shape the CRC immune microenvironment. We demonstrated that high METTL3 expression in CRC cells induced the m6A-BHLHE41-CXCL1 axis to promote the infiltration of myeloid-derived suppressor cells (MDSCs) in the CRC microenvironment, and that the loss of METTL3 in CRC compromised its ability to drive MDSC accumulation and suppressive potency, resulting in enhanced anti-tumor immune responses and diminished CRC growth [77]. In line with our study, a loss of METTL3 or METTL14 in CRC was reported to promote IFN-γ-Stat1-Irf1-signaling in an m6A-dependent manner to regulate anti-tumor immune responses upon anti-PD-1 therapy [78]. Intriguingly, the m6A eraser ALKBH5 displayed similar effects as the m6A writers METTL3 and METTL14. The knockout of ALKBH5 in CRC cells was capable of enhancing the efficacy of immunotherapy and prolonged mouse survival by promoting Mct4/Slc16a3 expression and increasing lactate content in the CRC microenvironment, which regulates the accumulation of Treg and MDSCs [82].

To characterize the m1A modifications pattern in CRC, Shi et al., conducted methylated RNA immunoprecipitation sequencing in pairs of human CRCs and adjacent normal tissues [98]. They identified different m1A distribution patterns of lncRNAs between CRC and adjacent tissues and pointed out that unique m1A distribution in CRC was correlated with several cancer pathways [98]. However, further study is warranted to explore the functional importance of m1A in CRC.

### 3.3. Gastric Cancer

Gastric cancer (GC) is the fifth most common cancer and the third most lethal malignancy worldwide [1]. Wang et al., reported that global RNA m6A level was increased in GC tissues compared to paired normal gastric mucosa which was attributed to elevated METTL3 expression [85]. A high METTL3 expression in GC tissues predicted poor prognosis of GC patients, implying that METTL3 is a potential prognostic factor [85]. Mechanistically, METTL3 promoted the m6A modification of HDGF mRNA and enhanced its mRNA stability, which was mediated by m6A reader IGF2BP3, thereby it promoted GC growth [85]. Corroborating its oncogenic function, METTL3 was reported to promote GC proliferation, migration, and invasion by regulating cancer-related pathways [86,99]. Notably, a high METTL3 expression promotes the chemoresistance of GC cells by inducing the m6A modification of ARHGAP5 to stabilize its mRNA expression [87].

Acting as m6A erasers, ALKBH5 and FTO are reported to promote GC tumorigenesis and progression. ALKBH5 demethylated the m6A marks of the lncRNA NEAT1 to promote GC metastasis [88]. On the other hand, FTO was capable of promoting GC growth and metastasis via repressing the m6A modification of caveolin-1 [90]. In this study, reduced m6A level of caveolin-1 mRNA promoted mRNA degradation, leading to the change in mitochondrial fission/fusion and metabolism [90]. FTO could serve as a poor prognostic risk factor for GC patients, and high FTO expression promoted GC cell migration and invasion by enhancing ITGB1 expression via suppressing its m6A level [89].

The m6A reader YTHDF1 was found mutated in around 7% of GC patients, and a high YTHDF1 expression predicted more aggressive GC progression and poor overall survival [92]. YTHDF1 acted as an oncogene in GC, and the depletion of YTHDF1 retarded GC growth both in vitro and in vivo [92]. Mechanistically, YTHDF1 could bind to m6A-modified FZD7 to promote its translation, leading to the activation of Wnt/β-catenin signaling [92]. Similarly, the m6A reader IGF2BP2 also played an oncogenic role in gastric carcinogenesis by inducing the IGF1R-RhoA-ROCK axis [91]. Our research team recently demonstrated that, in addition to the oncogenic role in GC cells, YTHDF1 could regulate the anti-tumor immune response through the repression of dendritic cells (DCs) [93]. Therefore, YTHDF1 may be a promising therapeutic target for GC treatment.

## 4. Targeting RNA Modification in Cancer

### 4.1. Inhibitors of RNA Modification Regulators

**Figure 2:**
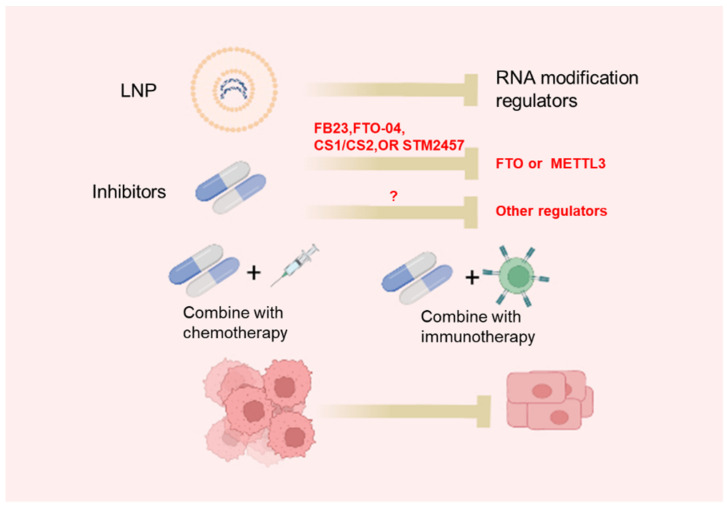
Approaches to target RNA modifications: LNP-siRNA or specific small molecular inhibitors targeting RNA modification regulators.

The dysregulated expression of m6A regulators as well as abnormal m6A profiles in cancer use epitranscriptomic inhibitors as a promising strategy for cancer treatment. Recently, more and more studies have focused on the development of inhibitors of m6A regulators (Table 4, Figure 2). UZH1a, a potent METTL3 inhibitor, is identified using a structure-based drug discovery approach [100]. UZH1a is selective and cell-permeable and could suppress the m6A methylation level of mRNA in several cell lines [100]. It is worth noting that the inhibitory effect of UZH1a against METTL3 activity is reported to last for at least 6 days [100]. STM2457 is another newly identified METTL3 inhibitor through a high-throughput screen of 250,000 diverse drug-like compounds [77]. STM2457 could selectively bind to the SAM site of METTL3 to suppress m6A catalytic activity with a half-maximal inhibitory concentration (IC50) of 16.9 nM [77]. Remarkably, the administration of STM2457 impaired acute myeloid leukemia (AML) growth and prolonged mouse survival in various mouse models of AML [101]. Thus, METTL3 is a new promising target for cancer therapy.

Given the functional significance of FTO in cancer, several FTO inhibitors have been developed. Using a structure-based rational design, two FTO inhibitors, FB23 and FB23-2 (derivatives of meclofenamic acid), have been constructed [103]. FB23 and FB23-2 could directly bind to FTO and suppress FTO-mediated demethylation without affecting ALKBH5 demethylation activity [103]. The administration of FB23 exerted a strong antileukemia effect in in vitro AML cell lines and patient-derived primary leukemia cells models, as well as in vivo patient-derived xenograft (PDX) mouse models [103]. Nevertheless, the IC50 values of FB23 and FB23-2 in suppressing AML are suboptimal: >20 μM and >1 μM for FB23 and FB23-2, respectively [103]. More recently, by a structure-based virtual screening of the 260,000 compounds, two compounds, named CS1 and CS2, were identified that displayed strong inhibitory effects against FTO activity [106]. CS1 and CS2 could strongly suppress AML cell viability with 10- to 30-fold lower IC50 [106]. Meanwhile, Liu et al., optimized FB23 and FB23-2 and developed Dac51 as a more potent FTO inhibitor [104]. Dac51 could strongly inhibit FTO demethylation activity with an IC50 of 0.4 mM [104]. Importantly, the administration of Dac51 was found to promote an anti-tumor T cell response and synergize with anti-PD-L1 treatment in suppressing melanoma [104].

RNA m1A modification is also a potential therapeutic target for cancer. Wang et al., identified thimerosal, phenylmercuric acetate (PMA), and thiram as inhibitors against the interaction of TRMT6 and TRMT61A via screening of 1600 known drugs [70]. TRMT61A/TRMT6 complex is responsible to catalyze RNA m1A modifications inside cells. Of these three drugs, the administration of thiram strongly reduced HCC growth in vivo, suggesting that targeting the TRMT6/TRMT61A complex is promising for HCC treatment [70].

### 4.2. Lipid Nanoparticles for In Vivo siRNA Delivery

The lipid nanoparticle (LNP) is among the most advanced nanocarriers that enable safe and effective siRNA delivery in vivo. LNP technology has been employed for treating various diseases in clinical practice [107,108,109,110]. The LNP delivery system could encapsulate siRNA targeting specific genes, enter cells through endosomes, and release siRNA into the cytoplasm [111]. For the successful delivery of nucleic acid into livers, LNPs are bound to ApoE protein which recognizes LDLR, a highly expressed receptor in hepatocytes [112,113]. Our team has recently utilized this platform to deliver siRNA targeting m6A-modified ARHGEF2 in mice CRC xenograft models. The results demonstrated that LNP-siARHGEF2 significantly suppressed tumor growth and metastasis in vivo, implying the therapeutic potential of LNP-siRNA for cancer patients [79]. However, the safety and efficacy of the LNP-siRNA targeting regulators of RNA modification for treating cancer should be further explored.

### 4.3. Targeting RNA Modification in Combination with Current Cancer Treatment

Resistance in therapy is a major challenge to the treatment of cancer patients. RNA modifications play a critical role in cancer development, providing novel insights into how to overcome treatment resistance. Moreover, the detection of RNA modification may serve as an indicator for individualized cancer treatment.

Dysregulated m6A modifications have been reported to regulate cancer chemotherapy resistance. A recent study revealed that the knockdown of FTO, an m6A eraser, could enhance the stem-like properties and chemo-resistance of CRC [83]. Intriguingly, the low expression of FTO in CRC was reported to promote acquired drug resistance through its N6,2′-O-dimethyladenosine (m6A_m_) demethylase activity [83]. Other m6A regulators have also been reported to influence the chemo-resistance of cancer. YTHDF3, an m6A eraser, was highly expressed in oxaliplatin-resistant CRC [114]. YTHDF3 could bind to eIF3A and form a complex to increase eIF2AK2 mRNA translation, leading to CRC chemotherapy resistance [114]. Another m6A reader IGF2BP3 could also increase CRC chemoresistance [115]. IGF2BP3 was reported to bind to m6A-modified ABCB1 mRNA, leading to enhanced mRNA stability, and high ABCB1 expression subsequently triggered the multidrug resistance of CRC [115]. Together, targeting m6A regulators may become of great utility to overcoming cancer chemotherapy resistance.

Targeting m6A regulators has shed new light to improve the effectiveness of cancer immune checkpoint inhibitor (ICI). Our recent study demonstrated that the targeting of METTL3 by METTL3-sgRNA or chemical METTL3 inhibitor STM2457 could potentiate the effect of anti-PD1 treatment in different mouse CRC syngeneic models [77]. Mechanistically, the targeting of METTL3 suppressed MDSC infiltration in a CRC microenvironment, which unleashed an anti-PD1-mediated CD8+ T cell antitumor response [77]. In line with our findings, Wang et al., reported that CRC cells with METTL3 or METTL14 knockout were more sensitive to anti-PD-1 treatment [78]. Therefore, METTL3 is a potential therapeutic target for combination therapy with ICI for CRC treatment.

Intriguingly, targeting m6A erasers ALKBH5 or FTO has also shown beneficial effects to enhance ICI efficacy for cancer treatment. By the in silico screening of a library of synthesized compounds, Li et al., identified a specific inhibitor of ALKBH5, named ALK-04 [82]. They demonstrated that the administration of ALK-04 strongly increased the efficacy of anti–PD-1 therapy response and prolonged the survival of mice bearing CRC allografts [82]. On the other hand, the knockdown of FTO was found to increase the sensitivity of melanoma cells to anti-PD-1 treatment in mice through the modulation of adaptive immunity [116]. Taken together, targeting m6A regulators could impact the ICI efficacy for CRC treatment.

## 5. Conclusions and Future Perspectives

RNA modifications have expanded our understanding of molecular mechanisms in GI cancer. However, there are many questions to be elucidated: (1) the change of RNA modifications at different stages of GI cancer development is still unclear; (2) the occurrence of RNA modifications among different types of RNA (mRNA, rRNA, tRNA, and lncRNA), or their positions among different regions of transcripts (5′UTR, CDS, and 3′UTR) shows the remarkable impact on the subsequent RNA metabolism, which should be further explored; (3) both oncogenic and tumor-suppressive functions of RNA modification regulators (e.g., METTL3) have been reported in cancer, reflecting their multifaceted roles during cancer development. This is partially contributed by the abundance of target transcripts while more evidence is required to address this issue; (4) the binding of different RNA modification readers could result in opposite outcomes. For example, YTHDF2 facilitates RNA degradation while IGF2BP1-3 stabilizes m6A-modified mRNAs. The discrepant results need to be justified; (5) the crosstalk or competition among different types of RNA modifications is largely unknown; (6) whether the detection of epitranscriptomic change could predict the treatment response of GI patients should be studied; and (7) although epitranscriptomic drugs have shown potential to promote the efficacy of chemotherapy or immunotherapy in preclinical studies, clinical trials should be designed and conducted in future. Overall, the complexity of epitranscriptome in GI cancer should be further studied, which will provide a novel therapeutic strategy for patients with GI cancer.

## Figures and Tables

**Table 1:** RNA modification and liver cancer.

Enzyme	Expression	Targets	Molecular Mechanism	Ref
METTL3	High	SOCS2	Silences SOCS2 by YTHDF2	[64]
METTL3	High	snail	SUMO1 modification of METTL3 promotes tumor progression	[65]
METTL3	High	snail	EMT transition	[66]
FTO	High	PKM2	Promote the proliferation and tumor growth	[67]
YTHDF2	Low	circCPSF6	Trigger circCPSF6 recognization and destabilization	[68]
YTHDF2	High	OCT4	promotes the liver cancer stem cell phenotype and cancer metastasis	[69]
TRMT61A/TRMT6	High	PPARδ	driving self-renewal of liver CSCs and tumorigenesis	[70]
METTL1	High	EGFR pathway	Enhances oncogenic mRNA translation and promotes intrahepatic cholangiocarcinoma progression	[71]
METTL1	High		promotes hepatocarcinogenesis via m 7 G tRNA modification-dependent translation control	[72]
METTL1		TGF-β2	promotes HCC recurrence after radiofrequency ablation	[73]
WDR4	High	CCNB1	promotes proliferation, metastasis, and sorafenib resistance	[74]

**Table 2:** RNA modification and colorectal cancer.

Enzyme	Expression	Targets	Molecular Mechanism	Ref
METTL3	High	GLUT1-mTORC1	Facilitates CRC growth	[75]
METTL3	High	miR-1246	Promotes cell migration, invasion, and metastasis	[76]
METTL3	High	BHLHE41-CXCL1	Induces immune suppression	[77]
METTL3/METTL14	High	Stat1 and Irf1	Regulate anti-tumor immune responses	[78]
YTHDF1	high	ARHGEF2	Promotes CRC growth	[79]
YTHDF2		GSK3β	Promotes CRC growth	[80]
YTHDC1	High	circNSUN2	Promotes CRC liver metastasis	[81]
ALKBH5	High	Mct4/Slc16a3	Regulate anti-tumor immune responses	[82]
FTO	Low	m6Am	Impedes CSC	[83]
FTO	High	MYC	Promotes CRC development	[84]

**Table 3:** RNA modification and gastric cancer.

Enzyme	Expression	Targets	Molecular Mechanism	Ref
METTL3	High	HDGF	Promotes GC growth, angiogenesis, and metastasis	[85]
METTL3	High	EMT markers	Promotes cell proliferation, migration, and invasion	[86]
METTL3	High	ARHGAP5-AS1	Promotes chemoresistance	[87]
ALKBH5	High	NEAT1	Promotes cell migration and invasion	[88]
FTO	High	ITGB1	Promotes GC metastasis	[89]
FTO	High	caveolin-1	Promotes GC growth and metastasis	[90]
IGF2BP2	High	IGF1R-RhoA-ROCK	Promotes GC progression	[91]
YTHDF1	High	FZD7	Promotes GC	[92]
YTHDF1	High		Promotes GC and induces antitumor immune response	[93]

**Table 4:** Inhibitors of RNA modification regulators.

Name	Target	Identification Methods	Cancer	Ref
UZH1A	METTL3	Using a structure-based drug discovery approach	leukemia	[100]
STM2457	METTL3	A high-throughput screen of 250,000 diverse drug-like compounds	leukemia	[101]
quercetin	METTL3	Drug screening	cervical	[102]
FB23/FB23-2	FTO	Using structure-based rational design	leukemia	[103]
Dac51	FTO	Optimized previously reported FTO inhibitors FB23 and FB23-2	melanoma	[104]
FTO-04	FTO	structure-based design	Glioblastoma	[105]
CS1/CS2	FTO	A structure-based virtual screening of the 260,000 compounds from NCI DTP library	leukemia	[106]
thimerosal, PMA, and thiram	TRMT6/TRMT61A	Screening of 1600 known drugs	HCC	[70]

## Data Availability

Not applicable.
